# Tracing the Invasion of *Takecallis nigroantennatus* (Hemiptera, Aphididae) on Cold-Hardy Bamboo *Fargesia* Using Mitochondrial COI Data

**DOI:** 10.3390/ijms26178608

**Published:** 2025-09-04

**Authors:** Karina Wieczorek, Dominik Chłond, Roma Durak, Matt Elliot, Anders Endrestøl, Jos Van der Palen, Beata Borowiak-Sobkowiak, Natalia Sawka-Gądek

**Affiliations:** 1 Institute of Biology, Biotechnology and Environmental Protection, Faculty of Natural Sciences, University of Silesia in Katowice, Bankowa 9, 40-007 Katowice, Poland; dominik.chlond@us.edu.pl; 2 Faculty of Biology, Nature Protection and Sustainable Development, University of Rzeszów, Pigonia 1, 35-310 Rzeszów, Poland; rdurak@ur.edu.pl; 3 Royal Botanic Garden Edinburgh, Edinburgh EH3 5LR, UK; melliot@rbge.org.uk; 4 Norwegian Institute for Nature Research (NINA), Sognsveien 68, NO-0855 Oslo, Norway; anders.endrestol@nina.no; 5 Bamboekwekerij Kimmei, 5555 LB Valkenswaard, The Netherlands; info@kimmei.com; 6 Department of Entomology and Environmental Protection, Poznań University of Life Sciences, Dąbrowskiego 159, 60-594 Poznań, Poland; 7 Institute of Systematics and Evolution of Animals Polish Academy of Sciences, Sławkowska 17, 31-016 Kraków, Poland; sawka@isez.pan.krakow.pl

**Keywords:** aphid, bamboo, founder effect, haplotype, insect, plant trade, population genetics

## Abstract

The introduction of alien insect species is increasingly facilitated by global plant trade, particularly through the movement of ornamental plants. *Takecallis nigroantennatus*, a host-specific aphid associated with cold-hardy *Fargesia* bamboo, has recently expanded its range in Europe. To examine its invasion dynamics, we conducted a population-level survey across 13 locations in six countries, sampling individuals from botanic and private gardens, specialized bamboo nurseries, garden centers, and urban horticultural environments in the UK, Belgium, The Netherlands, Germany, Poland, and Norway. A total of 117 specimens were analyzed using mitochondrial COI sequences, revealing a single dominant haplotype without geographic structure based on Bayesian and Maximum Likelihood phylogenetic analyses. This striking genetic uniformity indicates a narrow introduction bottleneck, suggesting a single or highly restricted introduction event followed by clonal spread. Despite the species’ ability for sexual reproduction, the data support a founder effect and rapid recent expansion closely linked to the introduction history of *Fargesia* in Europe. The results are also consistent with a possible time lag between the arrival of ornamental bamboo and the subsequent establishment of its associated herbivore, a scenario that warrants further investigation. Importantly, our study provides a practical framework for applied monitoring and early detection in bamboo nurseries, botanical gardens, and other high-risk introduction sites, illustrating how molecular tools can inform biosecurity and the management of emerging invasive species.

## 1. Introduction

Bamboo, a group of perennial grasses belonging to the subfamily Bambusoideae, is widely utilized across sectors such as food, textiles, construction, and environmental management. It also holds cultural, ecological, and growing economic significance in forestry and horticulture, particularly as a key element in the design of modern urban and private green spaces due to its aesthetic appeal, rapid growth, and ecological value [1]. Among the many genera cultivated in temperate regions, *Fargesia* species stands out as a cold-hardy, clump-forming bamboo, particularly suited to ornamental use in gardens across Europe and North America. Its non-invasive growth habit and evergreen foliage have contributed to its rising popularity in landscape architecture [2]. *Fargesia* was first introduced into Europe in the early 20th century by the British plant collector Ernest H. Wilson, who brought a single mother plant of *Fargesia murielae* from China. This species became popular in ornamental horticulture, but, as is typical of semelparous bamboos, many of these early plantings flowered simultaneously and subsequently died in the late 20th century. In response, a new wave of introductions occurred, including the documented import of *Fargesia* ‘Jiuzhaigou 1’ from Gansu Province, China, to Germany in the 1980s [3]. These new cold-hardy selections, especially *F. nitida*, *F. murielae*, and hybrid cultivars, were propagated in specialized nurseries, notably in The Netherlands, and began entering the wider horticultural market around the early 2000s. Since then, *Fargesia* cultivars have become increasingly available through commercial plant trade, as well as in institutional collections such as botanic gardens [4].

However, as the cultivation of ornamental bamboo expands, so too does the risk of unintentional introduction of associated herbivorous insects. Bamboos serve as host plants for a variety of insect taxa, particularly phloem-feeding hemipterans such as aphids. Several aphid species, primarily within the oriental genus *Takecallis* Matsumura (Hemiptera, Aphididae, Calaphidinae), have specialized trophic relationships with various bamboo taxa [5]. Recently, the hardy bamboo aphid *Takecallis nigroantennatus* Wieczorek, associated with *Fargesia*, was described based on specimens collected in Europe and distinguished from related species through both morphological and molecular characters [6,7]. This species, due to its feeding behavior, can negatively affect plant health and diminish the aesthetic value of ornamental *Fargesia* bamboo varieties, an important concern for horticultural and landscape applications [8].

The ornamental plant trade is a well-documented pathway for the unintentional movement of animals across continents [9,10]. The growing detection of *T. nigroantennatus* in non-native environments reflects broader global patterns of insect invasions, with horticultural trade in live plants being one of the primary pathways for alien species establishment [11,12]. Aphids are particularly suited to this type of dispersal due to their small size, parthenogenetic reproduction, short generation times, and ability to establish populations from a single viviparous female or overwintering egg. As herbivorous insects, aphids are strongly dependent on the availability of suitable host plants, which is a fundamental factor limiting the establishment success of non-native species. Consequently, several aphid species have been unintentionally introduced to new regions alongside their host plants [13,14,15], often establishing quickly under favorable conditions [16,17].

Despite its recent detection across several European countries, the invasion history of *T. nigroantennatus* remains unknown, largely due to the lack of data from its presumed native range in Asia and to the absence of a complete mitochondrial genome for this species. In this context, molecular tools have become indispensable in both species identification and the reconstruction of invasion pathways. The mitochondrial cytochrome c oxidase I (COI) gene is widely used in DNA barcoding due to its ability to discriminate between closely related species and detect cryptic diversity [18]. Beyond taxonomy, COI sequence data can be used to construct haplotype networks that reveal patterns of population structure, geographic differentiation, and historical dispersal. In invasive species research, such molecular approaches are essential for determining whether populations arise from single or multiple introduction events, assessing genetic bottlenecks, and evaluating the potential for adaptation in novel environments [19,20,21,22].

The aim of this study is to investigate the genetic structure of *Takecallis nigroantennatus* across its introduced range in Europe using mitochondrial COI barcoding. By analyzing individuals from multiple populations collected in six European countries, we assess the level of intraspecific variation, explore potential evidence of founder effects or clonal spread, and provide insights into the invasion dynamics of this emerging bamboo-associated aphid species. Additionally, this research contributes to documenting specific plant-insect invasion pairings and allows us to explore the possibility of a time lag between the introduction of ornamental bamboo species such as *Fargesia* and the establishment of specialized herbivores.

## 2. Results

### 2.1. Distributional Patterns and Potential Plant–Insect Time Lag

**Figure 1:**
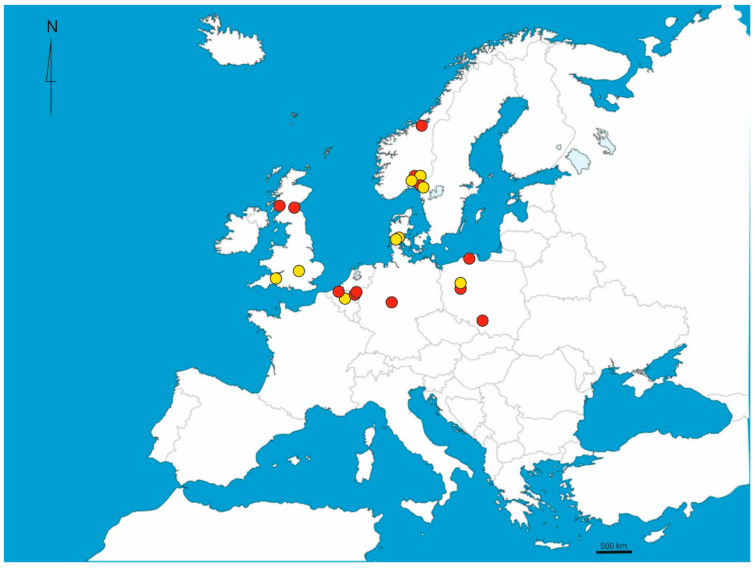
Current distribution of *Takecallis nigroantennatus* in its introduced range. Red dots indicate sampling locations from this study; yellow dots represent other known localities of the species.

Between 2023 and 2024, *Takecallis nigroantennatus* was collected from 13 sites across six European countries: the United Kingdom, Belgium, The Netherlands, Germany, Poland, and Norway. This represents the first coordinated Europe-wide survey of the species and includes several new country-level records. The presence of *T. nigroantennatus* in Germany, The Netherlands, Denmark and Norway is confirmed here for the first time. Notably, the northernmost record was obtained from Trondheim, Norway, extending the known range of the species into higher latitudes than previously reported (Figure 1).

**Figure 2:**
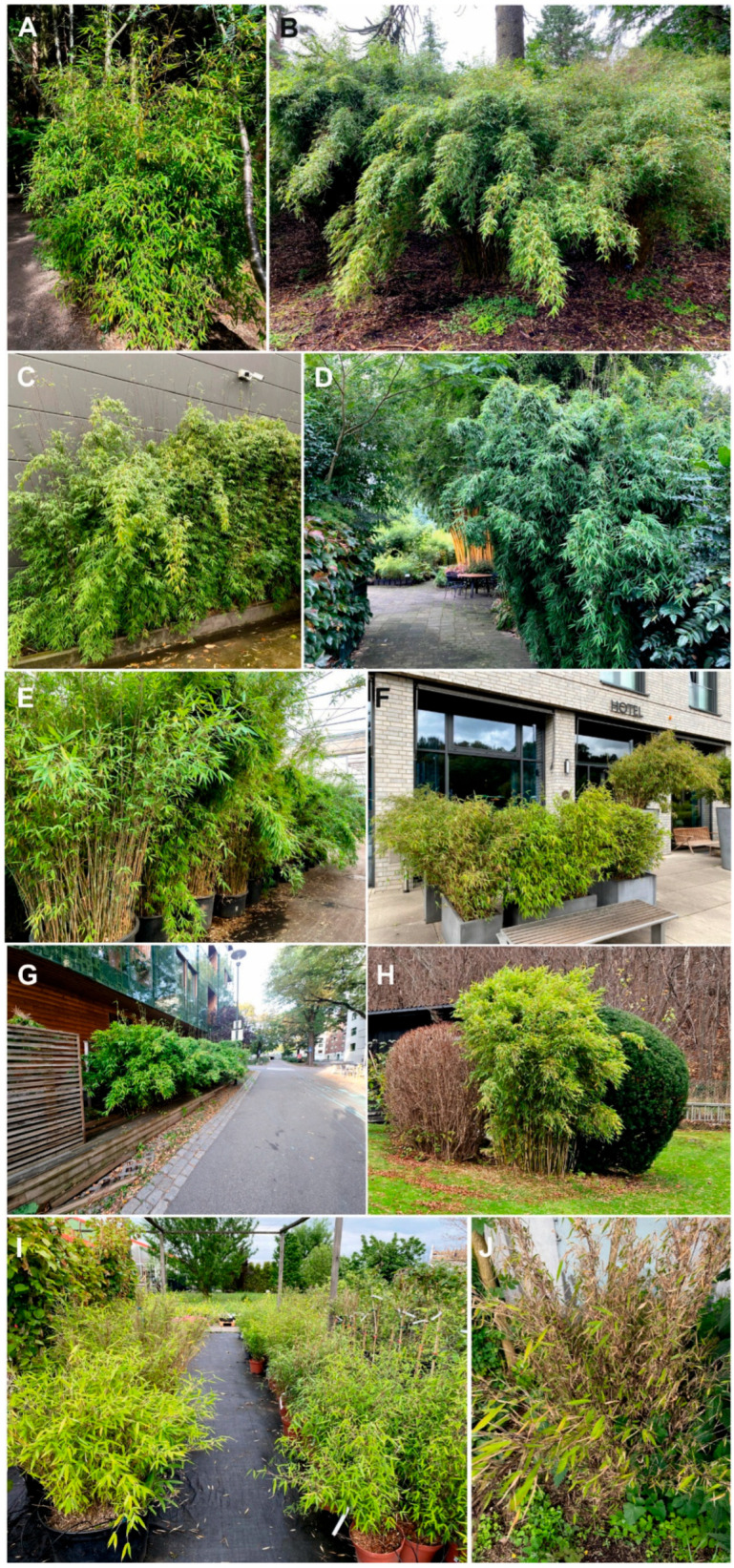
Sampling locations and host plants of *Takecallis nigroantennatus* in the introduced range. (**A**) *Fargesia nitida* in the RBG Edinburgh, UK. (**B**) *Yushania aceps* in the RBG Benmore, UK. (**C**) *Fargesia* sp. outside a garden center in Maldegem, Belgium. (**D**) *Fargesia* ‘Jiuzhaigou 1’ in Kimmei Nursery, The Netherlands. (**E**) *Fargesia nitida* ‘WinterJoy’ in Bamboo Giant Nursery, The Netherlands. (**F**) *Fargesia* sp. outside a hotel in Göttingen, Germany. (**G**) *Fargesia* sp. outside the Green House in Oslo, Norway. (**H**) *Fargesia* sp. in a private garden in Trondheim, Norway. (**I**) *Fargesia murielae* ‘Simba’ in a garden center in Siemianowice Śląskie, Poland. (**J**) *Fargesia* sp. in a private garden in Poznań, Poland.

Specimens were collected from a range of horticultural and semi-natural environments, including three botanic gardens (Edinburgh, Benmore, and Oslo), two specialized bamboo nurseries in The Netherlands (including Kimmei Nursery, which maintains the original European stock of *Fargesia* ‘Jiuzhaigou 1’), three garden centers (Belgium, Germany, and Poland), and several urban green spaces, such as hotel grounds, private gardens, and residential allotments. Aphids were found on at least seven different *Fargesia* taxa, including widely cultivated species such as *F. murielae* ‘Simba’, ‘Jumbo’, *F. nitida* ‘Winter Joy’, as well as on hybrid or unidentified *Fargesia* varieties. One population was collected from *Yushania aceps* in Benmore Botanic Garden (UK), a taxonomically related to *Fargesia* bamboo genus (Figure 2).

The chronology of host plant introduction and aphid detection suggests a time lag between the widespread cultivation of *Fargesia* and the recognition of its associated herbivore, *Takecallis nigroantennatus*. Cold-hardy *Fargesia* species, including *Fargesia* ‘Jiuzhaigou 1’, were introduced to Europe in the 1980s and became widely available through horticultural trade in Western Europe and the UK by the early 2000s. In contrast, *Fargesia* became commonly planted in countries such as Poland and Norway only within the last 10–15 years. The timing and pathway of *T. nigroantennatus* introduction remain uncertain, but it is plausible that the aphid arrived in Europe at some point during the expansion of *Fargesia* cultivation. Its morphological similarity to other *Takecallis* species may have contributed to its being overlooked or misidentified for years. Although first observed in the UK in 2015, the species was formally described in 2023 based on material from Poland. These patterns highlight the possibility of a prolonged but overlooked presence of the aphid in Europe, with more recent detections reflecting increased spread through trade and improved taxonomic resolution. However, in the absence of additional data such as retrospective identifications, museum entomological records, or comparative sampling from the native range, this time-lag scenario remains speculative.

### 2.2. Molecular Phylogeny and Network Analysis

Phylogenetic inferences were obtained for COI using Bayesian inference (BI) and Maximum likelihood (ML). The best-fitted model for COI was assessed using jModelTest to be GTR + G + I. ML and BI analyses resulted in phylograms with the same topologies. The Effective Sample Size (ESS) values reported for the BI analysis were >200, and the observed PRSF (Potential Scale Reduction Factor) was 1.0. Such a high ESS value indicates that the analysis reached convergence, the posterior distribution was sampled effectively, and the analysis produced estimated stable and reliable parameters. Phylogenies were solved, and the main splits were supported by high bootstrap values and posterior probabilities. Topologies were rooted using *Calaphis magnoliae* Essig and Kuwana and *Euceraphis lineata* Baker.

**Figure 3:**
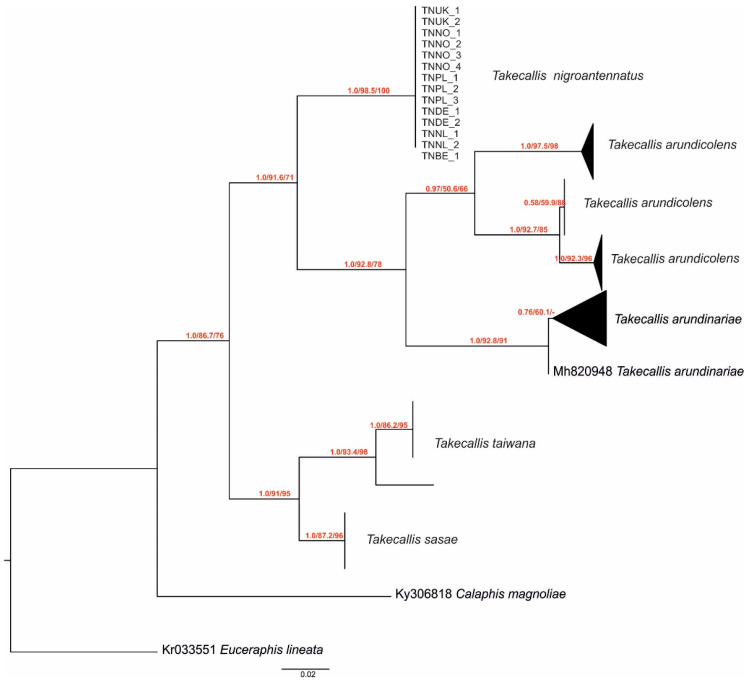
Phylogram displaying the phylogenetic position of all *Takecallis nigroantennatus* samples among *Takecallis* species, as determined by maximum likelihood (ML) and Bayesian inference (BI) analyses of cytochrome oxidase I (COI) gene sequences. Samples were collapsed to better visualize the phylogenetic tree. The first number on the branch is the posterior probability from MrBayes, the second is SH-aLRT support (%), and the third is the ultrafast bootstrap support (%) from IQ-TREE. Scale bar: expected substitutions per site.

The resulting phylogram revealed a well-supported clade formed exclusively by all *T. nigroantennatus* samples, indicating their clear genetic separation from other *Takecallis* species. In contrast, other species clustered into two additional clades: one composed of *T. sasae* (Matsumura) and *T. taiwana* (Takahashi), and the other including *T. arundicolens* (Clarke) and *T. arundinariae* (Essig) (Figure 3 and Figure S1).

Among the analyzed taxa, the mean pairwise distances (Table 1 and Tables S1–S3) showed no intraspecific variability in *Takecallis nigroantennatus* and also in *T. sasae.* Other species, *T. arundicolens* and *T. arundinariae,* show 3.7% and 0.5% intraspecific variability, respectively.

**Figure 4:**
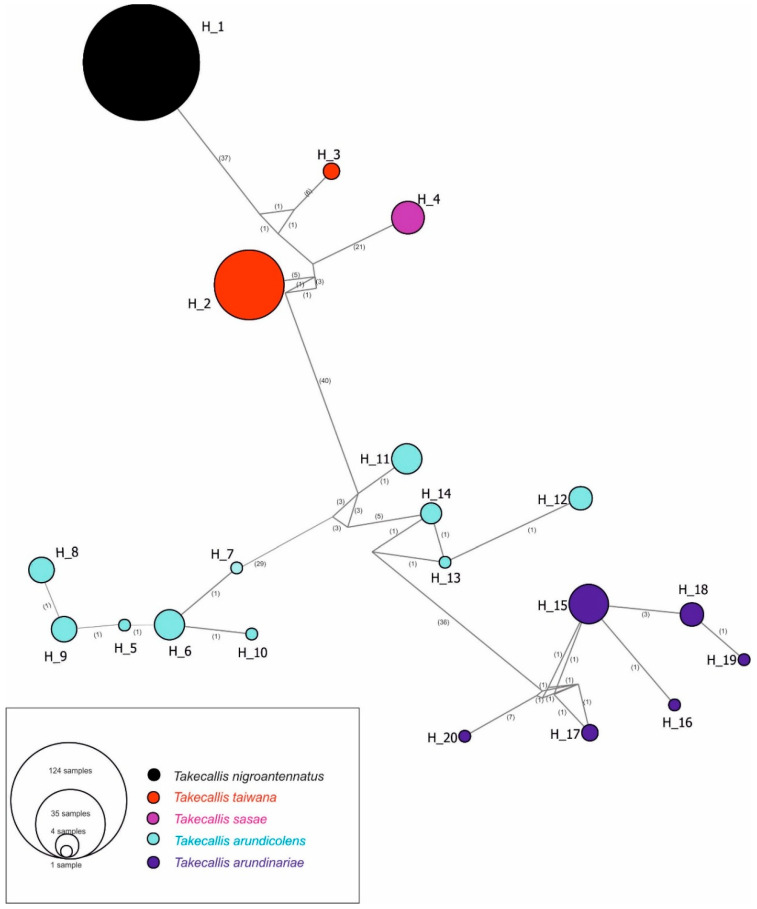
Median joining network of haplotypes for all analyzed *Takecallis* species. The colors represent the same species. The values on the nodes indicate the inferred number of variable sites between haplotypes.

Haplotype analysis based on the COI matrix identified a total of 20 haplotypes. Among these, a single haplotype (H1) was found for all *T. nigroantennatus* individuals analyzed, indicating no intraspecific variation within this dataset. For reference, the GenBank sequences included two haplotypes for *T. taiwana* (H2, H3), one for *T. sasae* (H4), ten for *T. arundicolens* (H5–H14), and six for *T. arundinariae* (H15–H20). No geographical clustering of COI haplotypes was observed for *T. nigroantennatus* or any of the reference species (Figure 4).

Overall, the phylogenetic tree and haplotype network both confirm the genetic coherence of *T. nigroantennatus* and its distinctiveness from other *Takecallis* species. The lack of haplotype diversity in *T. nigroantennatus* contrasts with the broader genetic variation observed among the reference taxa.

## 3. Discussion

Biological invasions are a major component of global environmental change, with widespread ecological, economic, and social consequences. They result from the successful establishment and spread of species outside their native ranges, facilitated by a combination of ecological traits, evolutionary processes, and, most critically, human activities such as trade, transport, and landscape modification. While some invasive species spread rapidly due to high propagule pressure or ecological flexibility, others may establish gradually or remain undetected for extended periods. Invasions can follow a variety of pathways, ranging from repeated introductions of genetically diverse individuals to single-introduction events involving very low genetic variation [23,24]. Thus, understanding these mechanisms and invasion stages is crucial for effective prevention, early detection, and the design of targeted management strategies [25,26].

Hemipteran insects are disproportionately represented among non-native arthropod introductions worldwide, largely due to their intimate relationships with live plants, high reproductive rates, and cryptic presence. These traits make them particularly well-suited to unintentional dispersal through the international plant trade [27]. The invasion pattern of *Takecallis nigroantennatus* in Europe, as revealed in this study, reflects a single-introduction, clonal expansion model. All analyzed populations from six European countries shared an identical COI haplotype, indicating a complete lack of mitochondrial diversity and pointing to a strong founder effect and genetic bottleneck. This suggests introduction via a narrow pathway, most likely through the ornamental bamboo trade, followed by rapid spread through clonal reproduction. The haplotype network confirmed the absence of regional genetic structure, further supporting the interpretation of a recent invasion derived from a single maternal lineage. Similar patterns of low genetic diversity have been reported in other invasive aphid species. *Sitobion avenae* (Fabricius), the cereal aphid, shows minimal mitochondrial and nuclear variation in Chilean populations, consistent with a strong founder effect and subsequent clonal expansion [28]. *Melanaphis sacchari* (Zehntner), the sugarcane aphid, shows extremely limited COI variation across its invasive range in the Americas and Africa, with populations often dominated by a single widespread “superclone” [29]. An even more extreme case is *Aphis nerii* Boyer de Fonscolombe, the oleander aphid, whose global populations are genetically uniform across both mitochondrial and nuclear markers [30]. These species are all anholocyclic, reproducing solely through parthenogenesis. In contrast, *T. nigroantennatus* is a holocyclic species capable of both asexual and sexual reproduction [8], yet it still exhibits no COI haplotype variation across Europe. This absence of mitochondrial diversity, despite the potential for recombination, further reinforces the interpretation of a recent, low-diversity introduction from a single maternal lineage followed by clonal spread.

Importantly, the observed genetic uniformity is not the result of geographically biased or ecologically limited sampling. Our dataset includes populations collected from a wide variety of horticultural and semi-natural environments, providing strong coverage of likely introduction and dispersal routes. We sampled *T. nigroantennatus* from two botanic gardens in the United Kingdom and one in Norway. We also confirmed its presence in the botanic gardens of Meise, Belgium [6], and Poznań, Poland [7], suggesting potential early introduction points via curated plant collections. Two samples of *T. nigroantennatus* were obtained from specialized bamboo nurseries in The Netherlands—one from *Fargesia* ‘Jiuzhaigou 1’ plant believed to be the first introduced to Europe and the other representing the largest bamboo nursery in Europe. In both cases, aphids were found on only a single plant, despite the presence of many *Fargesia* specimens. Similarly, no *T. nigroantennatus* individuals were detected in a comparable bamboo nursery in Belgium, where, as in the Dutch facilities, plants are carefully maintained and regularly monitored for pests. Further populations were obtained from garden centers in Belgium, Germany, and Poland, as well as from private gardens and urban greenery in Germany and Norway. The consistency of COI haplotypes across all these diverse sites confirms that the lack of variation is not an artifact of limited sampling but reflects a genuine genetic pattern linked to a narrow introduction history of the studied species.

At the same time, two important limitations should be acknowledged. First, the present dataset does not include specimens from the native Asian range of *T. nigroantennatus*. Without such reference material, it remains uncertain whether the European haplotype is unique to the introduced populations or part of a broader pool of unsampled diversity in East Asia. Comparative sampling will therefore be critical to fully reconstruct the invasion history and identify the precise geographic source of the European lineage. Second, our study relied exclusively on mitochondrial COI. This marker remains the most widely applied tool for DNA barcoding, haplotype identification, and broad-scale invasion genetics in aphids and other insects, and it has the advantage of being directly comparable to a large body of existing data. The absence of COI variation across Europe is therefore a robust finding and fits well with patterns reported in other invasive aphid species [28,29,30]. However, we acknowledge that COI alone cannot capture the full complexity of population-level processes in a holocyclic species. Nuclear markers such as microsatellites or SNP-based approaches would be needed to test for subtle structuring, quantify clonality in more detail, and explore whether additional diversity might exist at loci not captured by mitochondrial DNA. In this sense, the present study should be regarded as providing a genetic baseline: it documents the striking mitochondrial uniformity of European populations, but it also highlights the need for future multilocus or genomic studies to expand on these findings and refine our understanding of *T. nigroantennatus* invasion dynamics. More broadly, the study illustrates the value of applying even simple barcoding methods to newly established alien species. *T. nigroantennatus* is a recently described aphid that has only been recognized in Europe for a few years, yet it is already showing a rapid expansion across multiple countries. Establishing its first genetic profile, even with a single locus, is an important early step toward tracking its invasion history.

Our interpretation is further supported by the introduction pathway of its host plant, *Fargesia* ‘Jiuzhaigou 1’, which was brought to Europe in the late 1980s from a small number of seedlings collected in China. Initially propagated in Germany and later distributed through nurseries in The Netherlands, the UK, and beyond, *Fargesia* followed a narrow and well-documented route into European horticulture The first confirmed record of *T. nigroantennatus* in Europe occurred in the UK in 2015 (Buckinghamshire, and later in Llandaff and Cardiff), nearly three decades after the introduction of its host plant [31]. This temporal gap suggests a possible time lag between host establishment and aphid detection, which may be explained by delayed arrival, initial low-density populations, or misidentification with other *Takecallis* species known in Europe since the early 20th century. Notably, *Fargesia* species in the botanic gardens of Poznań and the Royal Botanic Garden Edinburgh have been growing in outdoor areas since the mid-1990s, supporting the possibility of long-term undetected presence of the aphid in such settings. While we cannot yet prove this scenario in the absence of comparative Asian data, it remains a plausible explanation that fits both the host plant history and the observed genetic uniformity. Future work incorporating wider sampling and genomic tools will be necessary to test whether *T. nigroantennatus* was established soon after *Fargesia* arrived in Europe, or whether its introduction occurred much later.

The tight ecological association between *T. nigroantennatus* and *Fargesia*, combined with the lack of genetic variation across sampled populations, underscores the critical need for improved monitoring of ornamental plant imports. Even in the absence of high genetic diversity, clonal insect invaders can spread widely and exert significant ecological or economic impact as documented in several aphid species exhibiting single “superclones” across invasive ranges [28,29,30], particularly when associated with popular, widely cultivated host plants. This concern aligns with broader trends highlighted by MacLachlan et al. [32], who demonstrated that the rate of Hemiptera introductions remains high despite strengthened biosecurity protocols and that many non-native species may remain undetected for years, a concept known as “establishment debt.” The case of *T. nigroantennatus* likely reflects such a scenario: a narrowly introduced, host-specific species that evaded early detection due to low visibility and taxonomic complexity, yet has expanded its range in parallel with the horticultural trade. Its spread also exemplifies the “hop-on, hop-off” dynamic, in which pest insects exploit the availability of suitable host plants across different stages of the plant trade and within horticultural environments [15]. New observational data further reinforce the establishment potential of *T. nigroantennatus*. In June 2025, heavy infestations were documented in two garden centers in Norway, Lier and Sarpsborg, on *Fargesia murielae* cultivars ‘Moontears’ and ‘Panda’, respectively, both originating from The Netherlands. A similar infestation was observed in July 2025 in a garden center of Kolding and private garden in Jels, both in Denmark, marking the first confirmed records of *T. nigroantennatus* in that country. Although not included in the COI analysis, these cases represent significant outbreaks and illustrate the continued movement of the aphid through the horticultural trade network. Additionally, in spring 2025, *T. nigroantennatus* was reconfirmed at previously recorded sites in Norway, including the Botanic Garden, Sogn Hageby, and Vika in Oslo, as well as in Ås, indicating persistent populations and local establishment. A similar infestation on *Fargesia* ‘Jiuzhaigou 1’ was also observed in a garden center in Siemianowice Śląskie, Poland, where the plants had been supplied by the same horticultural company as in the previous year’s observation. This repeated association suggests that plant distributors may act as a consistent source and pathway for the unintentional spread of *T. nigroantennatus* through the ornamental plant trade. However, it is worth noting that in early spring, aphid colonies may be easily overlooked due to low population density. In all known Norwegian and Polish locations, where follow-up surveys were conducted, aphid numbers remained relatively low during this period, with the exception of newly imported plants in garden centers.

Such findings highlight the importance of continued post-survey surveillance and underscore that new introduction events may go undetected without proactive monitoring. In this context, the principles of Early Detection and Rapid Response (EDRR) become especially relevant. Given the narrow introduction pathway of *T. nigroantennatus* and its strong association with ornamental cold-hardy *Fargesia* species, monitoring efforts should prioritize high-risk entry and dispersal points such as botanical gardens, specialized bamboo nurseries, and import facilities. Regular visual inspections of *Fargesia* plants, particularly recently imported or traded stock, should be complemented by targeted molecular surveillance to ensure early recognition of incipient populations. Even simple COI-based barcoding can be implemented in diagnostic laboratories to confirm species identity where morphological differentiation from congeners is difficult. Integration of these approaches into routine biosecurity monitoring would substantially improve the likelihood of rapid detection and containment of new incursions. Implementing molecular diagnostics at entry points, improving aphid taxonomic resolution during nursery inspections, and raising awareness among growers and landscape managers are key components of an effective EDRR framework to prevent unnoticed establishment and further spread of such invasive insects. A practical Biosecurity Protocol for both morphological and molecular identification of *T. nigroantennatus* is provided in Table S5, serving as a practical, ready-to-use tool for early detection and applied monitoring in botanical gardens, nurseries, and other high-risk introduction sites within the ornamental bamboo trade.

## 4. Materials and Methods

### 4.1. Sample Collection

A total of 117 specimens of *Takecallis nigroantennatus* were collected from 13 locations across six European countries: UK, Belgium, The Netherlands, Germany, Poland, and Norway. Sampling sites were selected to cover a broad range of potential introduction and dispersal pathways, including botanic gardens, specialized bamboo nurseries, garden centers, urban greenery, and private gardens. Specimens (adult winged viviparous females) were collected directly from the host plant—various ornamental *Fargesia* species and related bamboos (*Yushania*), reflecting the diversity of cultivated hosts in temperate Europe. Collection dates ranged from July 2023 to November 2024. All samples were preserved in 96% ethanol prior to molecular analysis. Detailed locality data, host plant identity, and sample metadata are presented in Table S4.

### 4.2. DNA Isolation, Amplification and Sequencing

DNA was extracted from 117 specimens from 13 locations across Europe. The population from Słupsk, Poland, where *T. nigroantennatus* was originally described, was also included in this study, with the corresponding COI sequence retrieved from GenBank, allowing direct comparison with newly collected material (Table S4). Genomic DNA was isolated without modifying the protocol using the Syngen DNA Mini Kit (Syngen, Wrocław, Poland). To elute the purified DNA, we applied 50 μL of an Elution Buffer onto the silica membrane. A fragment of the mitochondrial cytochrome c oxidase I (COI) gene was amplified with the primer pair LCO1490 and HCO2198 [33]. Polymerase chain reaction (PCR) amplification was performed in a final volume of 20 μL containing 30 ng of DNA, 10 μL of 2× Phanta Master Mix (Vazyme Biotech, Nanjing, China), 0.4 μL of 20 μM of each primer, in a Mastercycler ep system (Eppendorf, Hamburg, Germany). The cycling profile for the PCR was 95 °C for 3 min, 35 cycles of 95 °C for 15 s, Tm of oligos for 15 s, 72 °C for 1 min, and a final extension period of 72 °C for 5 min. In order to assess the quality of the amplification, the PCR products were electrophoresed in 1% agarose gel for 45 min at 85 V with a DNA molecular weight marker (Mass Ruler Low Range DNA Ladder, Thermo-Scientific, Waltham, MA, USA). The PCR products were purified using EPPiC (A & A Biotechnology, Gdańsk, Poland).

Samples were sequenced in both directions using the same primers as for the PCR reactions, combined with a BigDye Terminator 3.1 Cycle Sequencing Kit (Applied Biosystems, Waltham, MA, USA) in the chain termination reaction method [34]. The sequencing reaction was carried out with the PCR product at a total volume of 20 μL, containing 2 μL of BigDye Terminator Reaction Ready Mix v. 3.1, 2 μL 5× sequencing buffer, 3.2 mol/μL of the primer solution, and 6 μL of the purified PCR product. The cycle-sequencing profile was 3 min at 94 °C followed by 30 cycles of 10 s at 96 °C, 5 s at 50 °C, and 2 min at 60 °C. Sequencing products were precipitated using CleanDTR (CleanNa, Waddinxveen, The Netherlands), and were separated on an ABI PRISM 3130xl DNA Sequencer (Applied Biosystems, Waltham, MA, USA).

### 4.3. Sequence Edition and Alignment

Raw chromatograms were evaluated and corrected using Geneious v10.2.6 (https://www.geneious.com). To identify numts [35,36], the mitochondrial COI sequences were translated into amino acid sequences with Geneious v10.2.6 using the standard invertebrate mitochondrial genetic code and standard code. All nucleotide sequences were verified using BLAST v.2.16 searches of NCBI (http://blast.ncbi.nlm.nih.gov/Blast.cgi, accessed on 20 May 2025). The study sequences were aligned with other *Takecallis* species sequences obtained from GenBank using the MAFFT version 7.263 [37] plugin within Geneious v10.2.6. The alignment statistics was calculated in the DnaSP v.6 [38]. All sequences used for the analysis are listed in Table S4.

### 4.4. Phylogenetic Analysis and Haplotype Network Analysis

Models for Bayesian (BI) and maximum likelihood (ML) analyses were calculated in jModeltest [39] using the Akaike information criterion (AIC). BI analyses were carried out using MrBayes v3.2.6 [40] in four separate runs, each with three warm and one cold chain. Analyses were conducted for 500,000 generations, with trees sampled every 1000 generations. The first 25% of each run was discarded as burn-in. Convergence among the runs was assessed using Tracer [41], and examination of the potential scale reduction factor (PSRF) values and average standard deviation of the split frequencies in the MrBayes output was performed. The IQ-TREE online server [42] was used for ML analyses, including ultrafast bootstrap analysis and the aLTR SH-like test. All trees were visualized using FigTree (http://tree.bio.ed.ac.uk/software/figtree/, accessed on 10 June 2025). The number of haplotypes was calculated with DnaSP v.6 [38].

Pairwise distances were calculated using MEGA X with the p-distances model and the bootstrap method at 1000 repetitions (Table 1 and Table S1) [43]. Median-joining network of the obtained COI haplotypes were produced with Network v.10.2 (https://www.fluxus-engineering.com) [44] with default values.

The outline Europe map was obtained from FreeVectorMaps.com. Figures, trees, and network were edited and annotated in Corel Draw 17.1.0.572, 2014 Corel Corporation.

## 5. Conclusions

This study provides the first population-level genetic assessment of *Takecallis nigroantennatus* across its introduced range in Europe. The detection of a single COI haplotype among all analyzed individuals suggests that European populations likely originated from a single or highly restricted introduction event, followed by clonal expansion. This genetic uniformity is consistent with the known introduction history of *Fargesia* spp., particularly *Fargesia* ‘Jiuzhaigou 1’, which entered European horticulture through a narrow and well-documented pathway in the late 20th century.

While mitochondrial COI barcoding proved effective for broad-scale invasion pattern assessment and haplotype identification, several limitations should be acknowledged. COI represents a uniparentally inherited locus and may not reveal fine-scale population structure or hidden diversity. The absence of samples from the native range in East Asia prevents definitive identification of the geographic source of European populations. Higher-resolution nuclear markers, such as microsatellites or SNPs or whole-genome sequencing, would be necessary to quantify clonality, detect subtle population differentiation, and fully resolve the species’ introduction dynamics. Despite these limitations, the present COI dataset provides a valuable baseline for detecting founder effects, clonal spread, and lack of regional differentiation.

These findings emphasize the importance of integrating molecular data with historical, ecological, and horticultural information to reconstruct invasion history. They also highlight the need for targeted monitoring of ornamental bamboo trade and increased awareness among horticulturists and plant protection agencies. Future research should aim to expand geographic sampling to the native range of *T. nigroantennatus* and apply complementary nuclear markers to more comprehensively resolve population structure and invasion pathways. Such studies will improve early detection, biosecurity risk assessment, and long-term management of newly established aphid invasions, demonstrating that even initial single-locus assessments can provide actionable insights for invasive species management.

## Figures and Tables

**Table 1:** Mean p-distances within analyzed group of species based on COI sequences.

	p-Distance	Standard Error
*T. nigroantennatus*	0	0
*T. taiwana*	0.002	0.0004
*T. sasae*	0	0
*T. arundicolens*	0.0372	0.0055
*T. arundinariae*	0.005	0.0015

## Data Availability

The datasets presented in this study can be found in online repositories. The names of the repository/repositories and accession number(s) can be found at https://www.ncbi.nlm.nih.gov/, 23 June 2025: PV805314, PV805315, PV805316, PV805317, PV805318, PV805319, PV805320, PV805321, PV805322, PV805323, PV805324, PV805325, PV805326, PV805327, PV805328, PV805329, PV805330, PV805331, PV805332, PV805333, PV805334, PV805335, PV805336, PV805337, PV805338, PV805339, PV805340, PV805341, PV805342, PV805343, PV805344, PV805345, PV805346, PV805347, PV805348, PV805349, PV805350, PV805351, PV805352, PV805353, PV805354, PV805355, PV805356, PV805357, PV805358, PV805359, PV805360, PV805361, PV805362, PV805363, PV805364, PV805365, PV805366, PV805367, PV805368, PV805369, PV805370, PV805371, PV805372, PV805373, PV805374, PV805375, PV805376, PV805377, PV805378, PV805379, PV805380, PV805381, PV805382, PV805383, PV805384, PV805385, PV805386, PV805387, PV805388, PV805389, PV805390, PV805391, PV805392, PV805393, PV805394, PV805395, PV805396, PV805397, PV805398, PV805399, PV805400, PV805401, PV805402, PV805403, PV805404, PV805405, PV805406, PV805407, PV805408, PV805409, PV805410, PV805411, PV805412, PV805413, PV805414, PV805415, PV805416, PV805417, PV805418, PV805419, PV805420, PV805421, PV805422, PV805423, PV805424, PV805425, PV805426, PV805427, PV805428, PV805429, PV805430.

## Supplementary Materials

The following supporting information can be downloaded at https://www.mdpi.com/article/10.3390/ijms26178608/s1. References [45,46,47,48,49] are cited in the Supplementary Materials.
