# Supplementary material for: Tracing the Invasion of Takecallis nigroantennatus (Hemiptera, Aphididae) on Cold-Hardy Bamboo Fargesia Using Mitochondrial COI Data
Source: Int J Mol Sci. 2025 Sep 4;26(17):8608. doi: 10.3390/ijms26178608 (PMC12428861; doi:10.3390/ijms26178608)
Supplement: Supplementary file 1 [file ijms-26-08608-s001.zip › Supplementary material Legends.pdf]

Table S1: Statistic alignment.

Table S2: Estimated pairwise distances between species with standard error.

Table S3: Estimated mean pairwise distances between group of species with standard error.

Table S4: Data for aphid specimens used in this study. For each specimen, the table includes Sample population ID, DNA extraction voucher number (DNA Voucher number), collection data (locality, host plant, date of collection) and GenBank accession numbers for COI genetic marker.

Table S5: Practical biosecurity Protocol for detection of *Takecallis nigroantennatus* in the ornamental bamboo trade.

Figure S1: Maximum likelihood phylogenetic tree for all *Takecallis* species obtained using IQTree.
