# Supplementary material for: Tracing the Invasion of Takecallis nigroantennatus (Hemiptera, Aphididae) on Cold-Hardy Bamboo Fargesia Using Mitochondrial COI Data
Source: Int J Mol Sci. 2025 Sep 4;26(17):8608. doi: 10.3390/ijms26178608 (PMC12428861; doi:10.3390/ijms26178608)
Supplement: Supplementary file 1 [file ijms-26-08608-s001.zip › Supplementary Material Table S5.pdf]

Table S5: Practical biosecurity protocol for detection of *Takecallis nigroantennatus* in the ornamental bamboo trade.

This illustrated protocol provides guidelines for the rapid identification of *T. nigroantennatus* using both morphological characters and COI-based molecular methods. It is intended for use in botanical gardens, bamboo nurseries, import facilities, and research laboratories.

The hardy bamboo aphid *Takecallis nigroantennatus*		
STEP	ACTION	NOTES
1. Sample collection	Collect individual aphids from *Fargesia* cultivars	Use ethanol 70% for morphological and 95–100% for molecular ID; label site, name of host-plant and date
2. Morphological screening	Examine body color, antennae and cauda (Fot. 1-2).	Slide-mount winged adult viviparous female (Fot. 3) for accurate ID
3. DNA extraction	Use standard insect kits or rapid extraction	Single aphid sufficient for COI amplification
4. PCR amplification	Target COI gene with LCO1490/HCO2198 primers [Folmer et al. 1994]	PCR: 3 min at 95 °C followed by 35 cycles of 15 s at 96 °C, 15 s at 50 °C, 1 min at 72 °C and 5 min at 72°C
5. Sequencing & analysis	Sequence PCR product; compare with reference COI haplotype	Confirm 100% or near-identical match
6. Documentation & reporting	Record findings; preserve voucher specimens; document syptoms on host-plants (Fot. 4-6)	Integrate results into EDRR monitoring and quarantine records
7. Follow-up actions	Implement local containment or eradication measures	If necessary, alert relevant plant health authorities

Basic Information: *Takecallis nigroantennatus* is a holocyclic species. Winged viviparous females (Fot. 1) occur from April to November, while the sexual generation (wingless oviparous females and winged males, Fot. 2) appears between October and November. Winged morphs are bright canary-yellow with black antennae, except for segments I–II and the basal part of segment III. Oviparous females are characterized by brown dorsal sclerites and pale antennae. The cauda is always pale.

Dispersal occurs primarily through short-distance flight of winged adults and occasional long-distance dispersal by wind currents. However, the main pathway of spread is human-mediated, via the trade of commercially infested *Fargesia* cultivars. Infestation may not be immediately visible, as overwintering eggs remain concealed in the leaf sheaths from November to March.

Symptoms: Feeding by *T. nigroantennatus* causes visible changes on bamboo plants. Infested leaves may show yellowing, curling, or distortion, while heavy infestations lead to a general decline in plant vigor. Aphid feeding produces honeydew, which accumulates on leaf surfaces and supports the growth of sooty mold, leaving affected plants with a sticky and blackened surface. Colonies of the intensely yellow winged forms are often visible on both sides of leaves, especially during peak periods of population growth in spring/early summer and autumn (Fot. 4-6).

Methods of Detecting Presence: Detection of the pest relies on careful visual examination of bamboo plants. Both sides of the leaves, including the basal sheaths, should be inspected for signs of aphid feeding, honeydew, and the presence of different developmental stages of the insect. Plants showing symptoms should be examined more closely, and collected specimens may be submitted for laboratory analysis. Reliable identification requires either morphological study under a microscope (Fot. 3) or molecular confirmation using COI barcoding. Proper preservation and handling of samples are essential to ensure accurate diagnosis.

Local containment or eradication measures: When infestations of *Takecallis nigroantennatus* are detected, immediate containment is critical, particularly in nurseries, garden centers, or plant shops, where infested bamboo plants may otherwise be sold to customers who are unaware of the pest. Plants intended for sale should be carefully inspected and, if necessary, isolated before leaving the facility. Simple mechanical methods, such as washing plants with a strong jet of water, can effectively remove aphids and temporarily suppress colony growth. Biological control with natural enemies (e.g., lady beetles or lacewings) can further reduce pest pressure, especially in controlled environments. Only in cases of severe or persistent infestations should selective insecticides be considered, and their use should remain limited to avoid negative impacts on beneficial insects. Rapid removal of heavily infested plants from trade is especially important to prevent the unintentional spread of this species through the ornamental bamboo market. If infestations are detected in nurseries, garden centers, or import facilities, alert the relevant plant health authorities to ensure appropriate regulatory response and prevent further spread through trade.

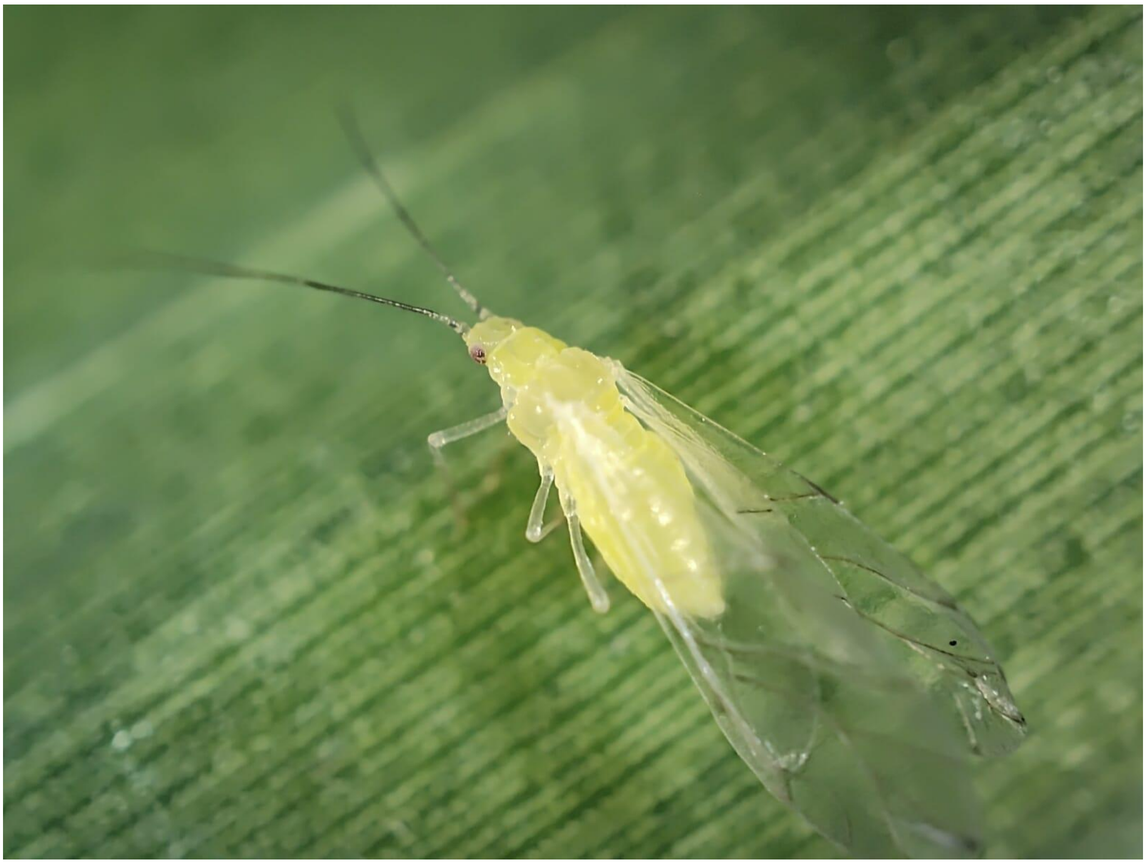

Fot. 1. Winged viviprous female of *Takecallis nigroantennatus*.

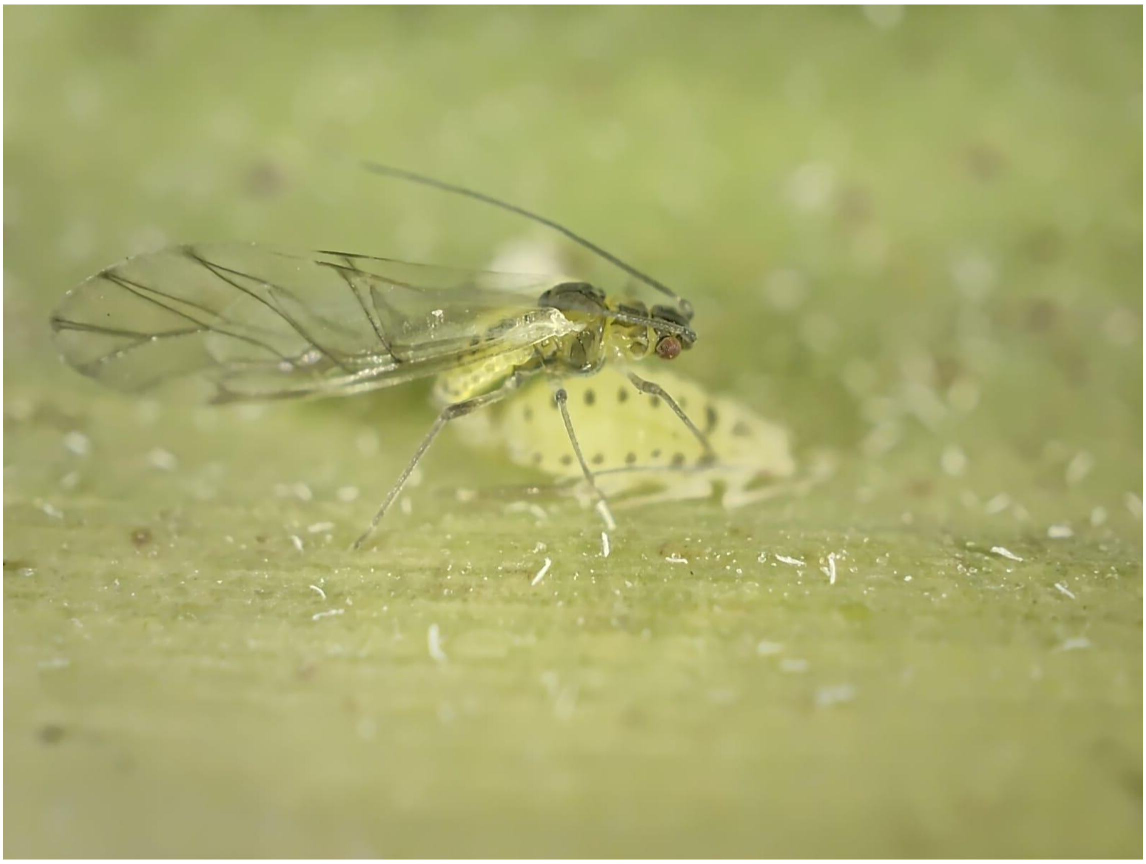

Fot. 2. Sexual generation of of *Takecallis nigroantennatus*.

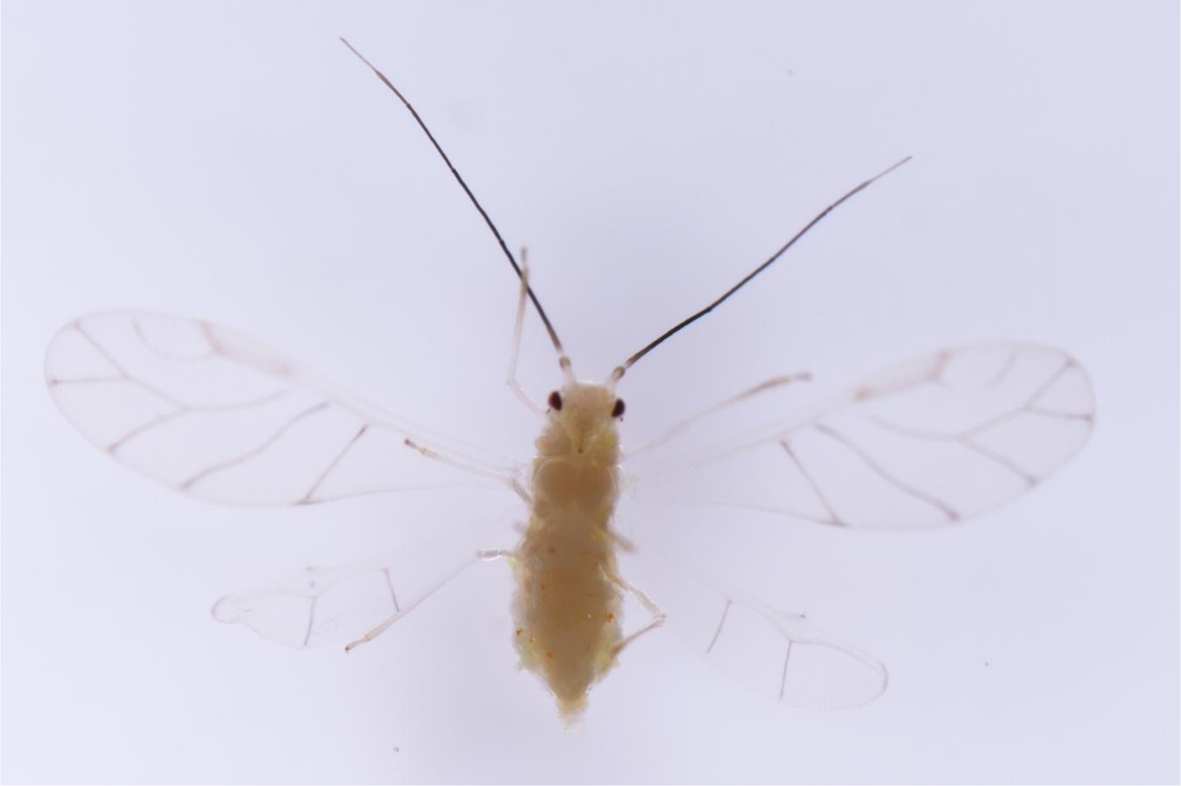

Fot. 3. Winged viviprous female of *Takecallis nigroantennatus* – microscopic slide ID.

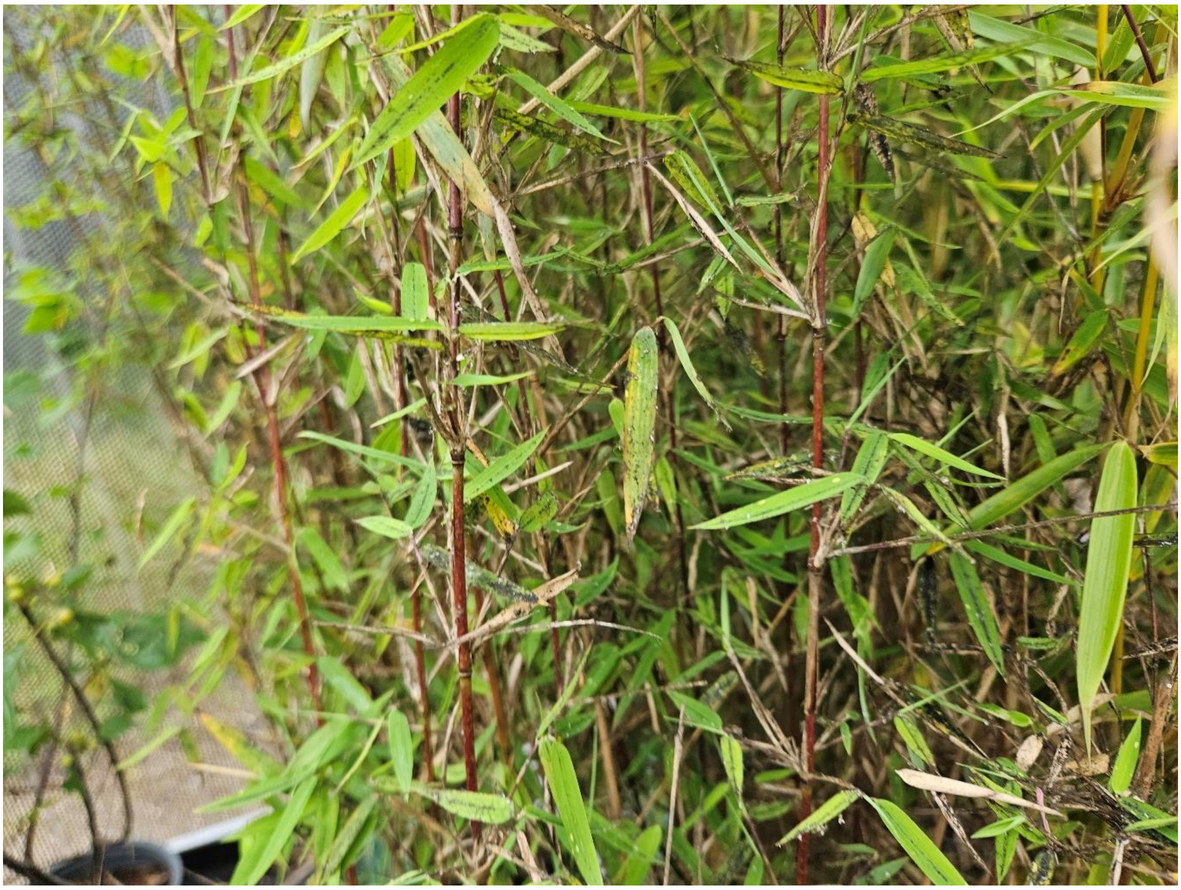

Fot. 4. Mass outbreak of *Takecallis nigroantennatus* on *Fargesia* ’Jiuzhaigou 1’ in a garden centre in Kolding, Denmark (summer).

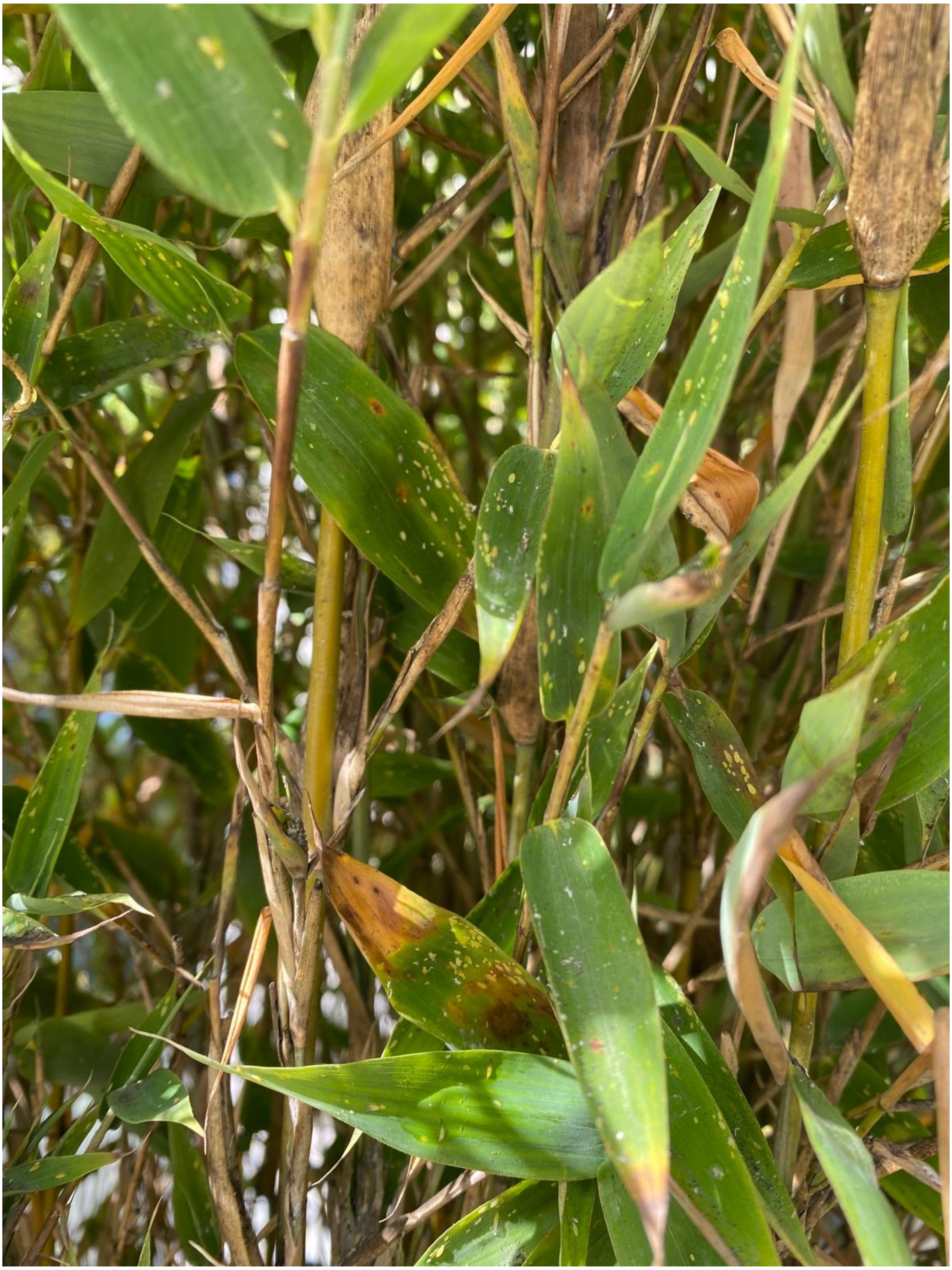

Fot. 5. Mass outbreak of *Takecallis nigroantennatus* on *Fargesia* sp. outside a hotel in Göttingen, Germany (autumn).

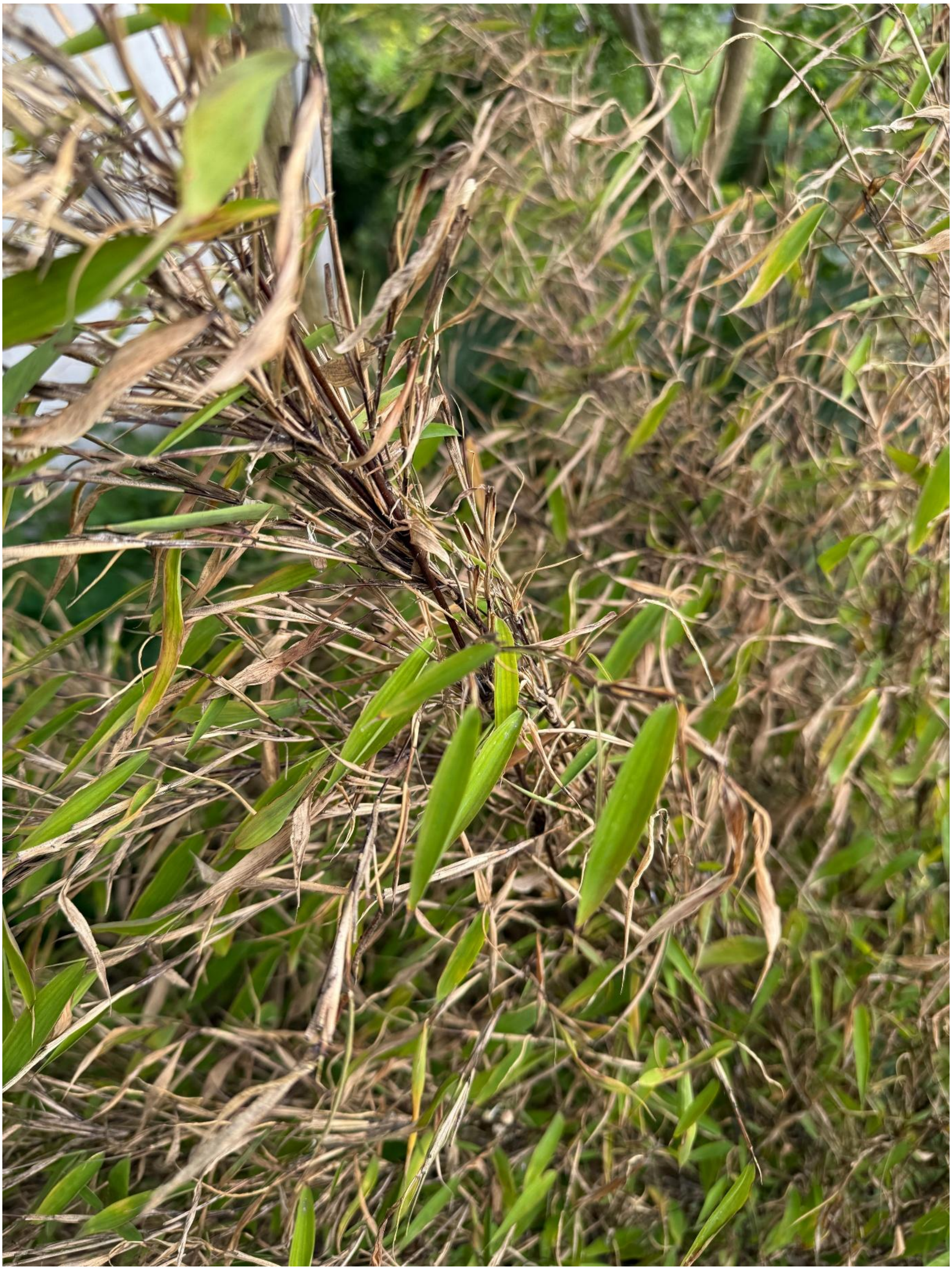

Fot. 6. Severe damage caused by *Takecallis nigroantennatus* feeding on *Fargesia* sp. after two years of cultivation in a private garden in Poznań, Poland (spring).
