# Supplementary figures and images for: Tracing the Invasion of Takecallis nigroantennatus (Hemiptera, Aphididae) on Cold-Hardy Bamboo Fargesia Using Mitochondrial COI Data
Source: Int J Mol Sci. 2025 Sep 4;26(17):8608. doi: 10.3390/ijms26178608 (PMC12428861; doi:10.3390/ijms26178608)

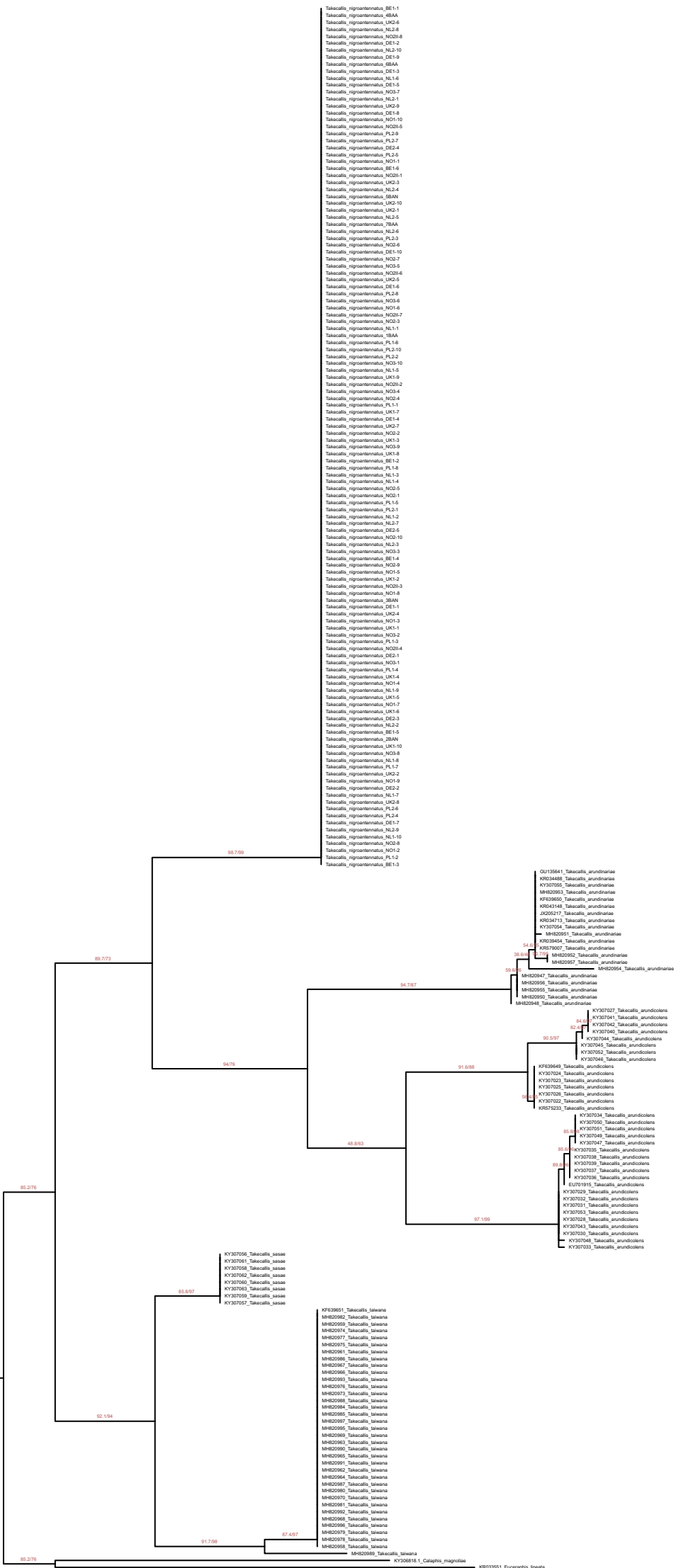

Supplement: Supplementary file 1 [file ijms-26-08608-s001.zip › Supplementary material Figure S1.pdf]
